# A novel method for monitoring abdominal compliance to optimize insufflation pressure during laparoscopy

**DOI:** 10.1007/s00464-022-09406-4

**Published:** 2022-07-21

**Authors:** Frank Sterke, Willem van Weteringen, Lorenzo Ventura, Ilaria Milesi, René M. H. Wijnen, John Vlot, Raffaele L. Dellacà

**Affiliations:** 1 Department of Pediatric Surgery, Erasmus MC Sophia Children’s Hospital, University Medical Center Rotterdam, Rotterdam, The Netherlands; 2 Department of Biomechanical Engineering, Delft University of Technology, Delft, The Netherlands; 3 Dipartimento di Elettronica, Informazione e Bioingegneria, Politecnico di Milano University, Milan, Italy

**Keywords:** Endoscopic oscillometry, Abdominal compliance, Surgical workspace, Insufflation pressure, Laparoscopy, Individualized pneumoperitoneum

## Abstract

**Background:**

Abdominal compliance describes the ease of expansion of the abdominal cavity. Several studies highlighted the importance of monitoring abdominal compliance (C_ab_) during the creation of laparoscopic workspace to individualize the insufflation pressure. The lack of validated clinical monitoring tools for abdominal compliance prevents accurate tailoring of insufflation pressure. Oscillometry, also known as the forced oscillation technique (FOT), is currently used to measure respiratory mechanics and has the potential to be adapted for monitoring abdominal compliance. This study aimed to define, develop and evaluate a novel approach which can monitor abdominal compliance during laparoscopy using endoscopic oscillometry.

**Materials and methods:**

Endoscopic oscillometry was evaluated in a porcine model for laparoscopy. A custom-built insufflator was developed for applying an oscillatory pressure signal superimposed onto a mean intra-abdominal pressure. This insufflator was used to measure the abdominal compliance at insufflation pressures ranging from 5 to 20 hPa (3.75 to 15 mmHg). The measurements were compared to the static abdominal compliance, which was measured simultaneously with computed tomography imaging.

**Results:**

Endoscopic oscillometry recordings and CT images were obtained in 10 subjects, resulting in 76 measurement pairs for analysis. The measured dynamic C_ab_ ranged between 0.0216 and 0.261 L/hPa while the static C_ab_ based on the CT imaging ranged between 0.0318 and 0.364 L/hPa. The correlation showed a polynomial relation and the adjusted R-squared was 97.1%.

**Conclusions:**

Endoscopic oscillometry can be used to monitor changes in abdominal compliance during laparoscopic surgery, which was demonstrated in this study with a comparison with CT imaging in a porcine laparoscopy model. Use of this technology to personalize the insufflation pressure could reduce the risk of applying excessive pressure and limit the drawbacks of insufflation.

The primary goal of insufflation is the creation of laparoscopic workspace, for which the abdominal cavity is distended by insufflation of pressurized carbon dioxide gas. In laparoscopy, both the benefits of a well-exposed surgical field [1, 2] and the negative consequences of high insufflation pressures [3, 4] have been thoroughly investigated. Sufficient surgical workspace results in a shorter procedure duration and reduces complications [5]. The application of high pressures impairs organ perfusion, hampers mechanical ventilation and results in postoperative pain and delayed recovery [6, 7]. For optimal surgical conditions, the insufflation pressure setting should be based on whether the benefits of gaining workspace volume outweigh the drawbacks of applying a higher insufflation pressure [8, 9]. This trade-off can only be assessed after acquiring access to the created pneumoperitoneum, depends on the patient-specific ease of abdominal expansion [10–12] and is affected by several biomechanical and pharmacological factors [13, 14].

The elastic behaviour of the abdominal cavity determines the shape of the pressure–volume (P–V) relationship of the laparoscopic workspace [15]. The P–V curve has a non-linear shape: at lower insufflation pressures the abdomen expands easily and each pressure increment results in larger volume gains. At higher insufflation pressures, the ease of abdominal expansion reduces and further pressure increments will provide diminishing gains in intra-abdominal volume [11, 15–18]. The slope of the P–V curve indicates the ease of expansion and is known as abdominal compliance (C_ab_) [19]. The reduced C_ab_ at higher insufflation pressures implies that disproportional stress is applied to the surrounding tissues and organs, without significant improvement of surgical workspace. During creation of the pneumoperitoneum, the tension of the abdominal wall can be assessed. However, this only provides limited insight into the tension/stress exerted onto the internal tissues and organs. Despite the evident need for monitoring the relation between insufflation pressure and the resulting workspace during laparoscopy, there is no practical method available for clinical use [19].

Availability of real-time measures of C_ab_ can be useful for finding an individual trade-off for insufflation pressure. Several studies highlighted the importance of monitoring C_ab_ during insufflation to personalize the intra-abdominal pressure (IAP) to create the largest workspace volume that does not compromise patient safety [10, 19, 20]. Unfortunately, measuring C_ab_ imposes relevant technical challenges: when using a conventional insufflator, measuring the insufflated CO_2_ gas volume cannot provide an accurate estimation of the surgical workspace volume, as gas leaks out at the trocars or is removed through suction, while CO_2_ is also absorbed by the peritoneum over time. The only available methods that can accurately measure laparoscopic workspace rely on volumetric imaging techniques, such as computed tomography (CT) or magnetic resonance imaging (MRI) [20]. These techniques, however, are too cumbersome and invasive for routine intraoperative monitoring.

Oscillometry, also known as the forced oscillation technique, is potentially a practical method for intraoperative monitoring of C_ab_ and is an established method for assessing the mechanical properties of the respiratory system [21–23]. Oscillometry applies a small-amplitude and high-frequency oscillating pressure signal at the airway opening. The resulting oscillatory flow is used to determine the mechanical impedance of the respiratory system, which is then used to estimate respiratory system compliance. The main advantage of oscillometry is that it can be applied during spontaneous breathing [23].

Theoretically, the C_ab_ can be assessed during laparoscopy using a similar approach. In this case, the insufflator generates the small-amplitude pressure oscillations superimposed onto the IAP. The compliance of abdominal tissues determines the resulting oscillatory gas flow, which can be measured with a flow sensor in the insufflation circuit. The abdominal mechanical impedance (Z_ab_) is determined by decomposing the pressure and flow into their oscillatory components and taking the ratio between the two. A mathematical model describes the dynamic behaviour of the abdomen and uses Z_ab_ to calculate C_ab_ in L/hPa.

This study aimed to define, develop and evaluate a system that uses endoscopic oscillometry to determine abdominal compliance. The abdominal compliance estimated with this custom-built system was validated against compliance calculated from CT scans, obtained at a range of insufflation pressures in a porcine model for laparoscopy.

## Materials and materials

### Animals

The female Landrace pigs, with an approximate weight of 20 kg, which were included in this study were part of a larger study protocol. In this protocol, animals were randomized in groups for deep, moderate or no neuromuscular blockade (NMB). To reflect the common clinical practice of using NMB and to rule out the effect of diaphragmatic muscle activity in affecting pressure and volume measurements only animals with deep NMB were included in this study. Deep muscle relaxation was titrated to a post tetanic count below 1/10. During their accommodation period, the pigs had free access to food and water and were provided enriched housing. On the day of the experimental procedure, the pigs only had access to water. Animals were excluded when anatomical deformations were found that could affect cardiorespiratory physiology. The overarching study protocol was registered under license number AVD101002015180 at the Dutch Central Authority for Scientific Procedures on Animals. Institutional approval was given by the Animal Welfare Body of Erasmus MC, University Medical Center Rotterdam, protocol number 15-180-02,2,1.

### Animal preparation

Pigs were pre-anaesthetized with an intramuscular injection containing ketamine, midazolam and atropine. Consequently, they were placed in a supine position, intubated and connected to the mechanical ventilator (fabian HFO, Acutronic Medical Systems AG, Hirzel, Switzerland). All animals were mechanically ventilated using volume guarantee mode. In line with clinical guidelines [24], mechanical ventilation was set to provide a tidal volume of 7.5 mL/kg with a positive end-expiratory pressure (PEEP) of 5 cmH_2_O (3.75 mmHg). General anaesthesia was maintained by intravenous administration of propofol (14 mg/kg/h), sufentanil (6.5 mg/kg/h). Rocuronium was started at 8 mg/kg/h and titrated to the desired deep level of NMB.

For insufflation, a 12 mm trocar was placed at the lower midline (VersaOne, Medtronic, Fridley, USA). Uncomplicated intraperitoneal trocar placement was verified endoscopically. During the animal preparation and experimental protocol, mechanical ventilation settings were adapted to maintain adequate oxygenation and ventilation. Oxygenation was managed by adapting the fraction of inspired oxygen, ventilation was adjusted by changing the respiratory rate.

### Experimental protocol

To cover a large range of abdominal compliances, each animal underwent a stepwise insufflation protocol in which the insufflation pressure was set to 5, 8, 10, 12, 14, 16, 18 and 20 hPa (3.75, 6, 7.5, 9, 10.5, 12, 13.5 and 15 mmHg). Although clinically 5 and 8 hPa usually do not provide sufficient surgical workspace, these pressures were included to adequately describe the full compliance curve. At each pressure level, the insufflation pressure was maintained for 3 min to allow volume stabilization and adaptation of tissues to the changed IAP. At each pressure level, measurements were taken during an end-expiratory pause. During this pause, the mechanical ventilator provided a continuous positive airway pressure equal to the PEEP of 5 cmH_2_O (3.75 mmHg).

### Measurements

*Intra-abdominal volume* At every step, a CT scan with a 1 mm slice thickness was obtained using a Somatom Force scanner (Siemens Healthcare GmbH, Erlangen, Germany). From every CT scan, the intra-abdominal CO_2_ volume (IAV) was calculated using Myrian imaging software (Intrasense, Montpellier, France).The segmentation resulted in volume measurements with a 0.001 L accuracy.

*Endoscopic oscillometry recording* To assess the mechanical impedance of the pneumoperitoneum, a sinusoidal pressure signal was superimposed onto the mean insufflation pressure.

**Fig. 1:**
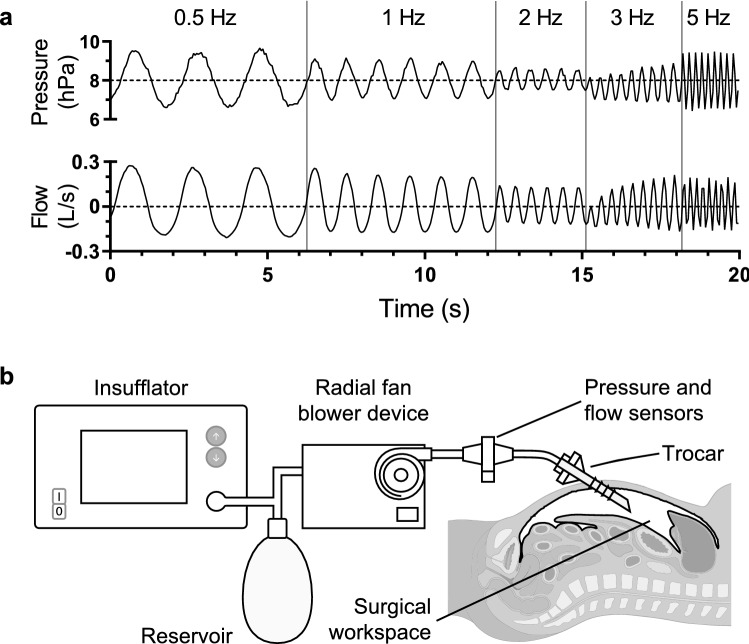
Endoscopic oscillometry sequence and overview measurement setup. **a** The time course of insufflation pressure and trocar flow during endoscopic oscillometry (─). Mean insufflation pressure and trocar flow (- -). **b** Endoscopic oscillometry measurement setup

For the endoscopic oscillometry recording, a custom-built turbine-based device generated sinusoidal pressure signals with a peak-to-peak amplitude of 3 hPa (2.25 mmHg) superimposed onto the set IAP. For each endoscopic oscillometry recording, there is a trade-off between the applied frequency and the time needed to perform the measurements. The device was programmed to apply a sequence of 4 to 10 sinusoidal cycles at 0.5, 1, 2, 3 and 5 Hz, leading to an overall signal duration of 20 s. Figure 1a shows an example of a recorded endoscopic oscillometry sequence. Pressure and flow were measured using differential pressure transducers connected to a mesh-type pneumotachograph (HCLA Series, First sensor AG, Berlin, Germany and PNT 8410A, Hans Rudolph Inc., Shawnee, USA).

The schematic overview of the measurement setup and custom-built insufflation device for generating the endoscopic oscillometry sequence is shown in Fig. 1b. The pneumotachograph was connected to the main lumen of the trocar via a 40 cm tube with a lumen diameter of 9 mm. Pressurised CO_2_ was supplied by a commercially available insufflator (Endoflator 40 UI, Karl Storz GmbH & CO. KG, Tuttlingen, Germany) to a 4 L reservoir consisting of two anaesthesia reservoir bags (2 L reservoir bag, Intersurgical Ltd., Berkshire, UK). The gas in the reservoir was used to constantly feed a radial fan blower (U65MN-024KD-5, Micronel AG, Tagelswangen, Switzerland) driven by a servo controller (Escon, Maxon motor AG, Switzerland) to produce the combination of a constant IAP and sinusoidal pressure signals. The pressure signal for the endoscopic oscillometry sequence was controlled via a closed-loop control system that would adapt the radial fan blower speed based on the measured pressure signals. A microcontroller (CY8C5888LTI-LP097, Cypress Semiconductor Corp., San Jose, USA) mounted on a custom electronic board acquired the signals from the transducers and controlled the IAP and endoscopic oscillometry sequence at 1000 Hz. The pressure and flow transducers were calibrated before and after each experiment. The pressure was calibrated using a reference calibration device (IMT Analytics FlowAnalyser PF-300, IMT Analytics AG, Buchs, Switzerland), the flow transducer was calibrated using a 100 mL calibration syringe (Series 5510, Hans Rudolph Inc., Shawnee, USA). Both pressure and flow data were sent to a laptop with a sampling rate of 200 Hz via a serial interface for subsequent offline data analysis.

### Data analysis

#### Static abdominal compliance

The IAV was used to calculate the static abdominal compliance (C_ab,stat_). Specifically, to determine C_ab,stat_ for a given insufflation pressure, the slope of the pressure–volume curve at that pressure level needs to be estimated. In this study, the slope of the pressure–volume curve was calculated as the pressure derivative of a pressure–volume equation able to fit the experimental data. Equation 1 was empirically found to describe the relationship between IAV and insufflation pressure *p:*

1 $$IAV\left(\mathrm{p}\right)={\mathrm{IAV}}_{\mathrm{max}}- \frac{{\mathrm{IAV}}_{\mathrm{max}}}{{\mathrm{e}}^{\uplambda \cdot \left(\mathrm{p}-{\mathrm{p}}_{0}\right)}}$$

This equation uses three parameters: a baseline pressure (p_0_); the maximum IAV which is obtained at an infinitely high-pressure (IAV_max_) and the expansion rate (λ). C_ab,stat_ was obtained by differentiation the equation for IAV with respect to *p*:

2 $${\mathrm{C}}_{\mathrm{ab},\mathrm{stat}}\left(\mathrm{p}\right)=\uplambda \cdot \frac{{\mathrm{IAV}}_{\mathrm{max}}}{{\mathrm{e}}^{\uplambda \cdot \left(\mathrm{p}-{\mathrm{p}}_{0}\right)}}$$

Equation 1 was fitted onto measured IAV using the least-squares method. The quality of fit was assessed by calculating the root mean squared error (RMSE). Using the obtained parameters for each subject, Eq. 2 was used to calculate C_ab,stat_ for every IAP.

#### Dynamic abdominal compliance

The C_ab,dyn_ was estimated from the recorded insufflation pressure and flow by analysing the abdominal impedance (Z_ab_) using an RLC-model to determine resistance (R in hPa s/L), inertance (commonly abbreviated as L in hPa s^2^/L) and compliance (C in L/hPa). Z_ab_ was calculated by taking the complex ratio between the Fourier transforms of pressure and flow. The impedance curve was created by calculating the Z_ab_ for each of the applied frequencies. For each frequency segment, Z_ab_ was computed using Welch periodogram averaging [25]. The Welch averaging window length was double the period of the sinusoidal wavelength, with a 50% overlap between segments. Onto every Welch averaging segment, a Hamming window was applied to minimize Fourier transformation errors [26]. The quality of the estimated Z_ab_ was assessed by calculation of the magnitude squared coherence. The signal coherence indicates the signal quality by examining the strength of the relation between the applied pressure oscillations and the resulting measured gas flow. The Z_ab_ was determined for every frequency and the RLC-model was curve fitted onto this data using the least-squares method. Impedance is expressed as a complex number with a real and an imaginary component, in graphs these components are usually presented separately. The RLC-model describes the Z_ab_ in terms of resistance, inertance and compliance. In the case of abdominal impedance, capacitance is a measure of storage of gas volume and equivalent to the C_ab,dyn_. The resistance describes the real component of Z_ab_, which is a measure of energy losses that can occur in the insufflation circuit or due to deformation of the abdominal cavity. The inertance describes inertial properties and is a measure of the pressure needed to accelerate the CO_2_ gas within in the insufflation circuit. The accuracy of the RLC-model curve fit was evaluated using the calculated RMSE.

#### Comparing static and dynamic abdominal compliance

To validate the ability of endoscopic oscillometry to estimate C_ab_, CT volume measurements were used as the golden standard for determining C_ab_. Therefore C_ab,stat_, was compared against the C_ab,dyn_ from endoscopic oscillometry measurements. The relationship between C_ab,stat_ and C_ab,dyn_ was described using second-order polynomial regression. The validity of the relationship was tested by calculating the adjusted R-squared.

## Results

### Intra-abdominal volume measurements and static compliance

**Fig. 2:**
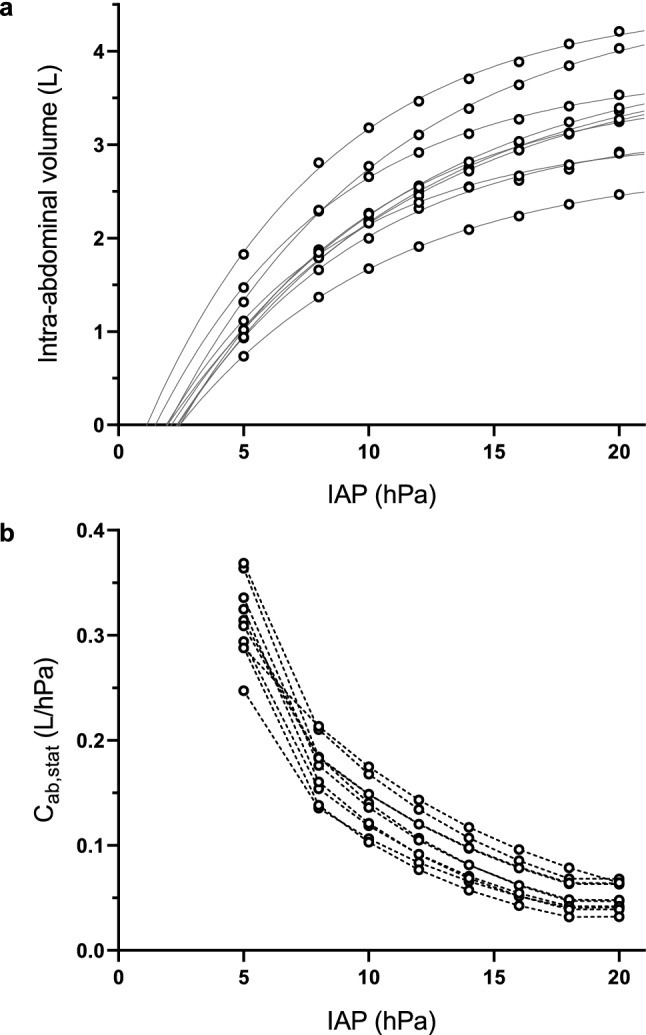
Abdominal pressure–volume curves and estimated static abdominal compliance. **a** Intra-abdominal volume vs IAP, measured IAV (○) and curve fit (─). **b** Estimated static abdominal compliance vs IAP (-○-)

Eleven animals were investigated in this study, one animal was excluded due to anatomical abnormalities. The median body weight was 21.4 kg and the IQR was between 19.6 kg and 22.2 kg. A total of 80 CT scans were analysed to measure the abdominal cavity volume at the different levels of IAP. Figure 2a shows the measured intra-abdominal pressure-volumes and the curve fitting provided by Eq. 1. At an IAP of 20 hPa, IAVs ranged between 2.47 L and 4.21 L. Within every subject, the IAV increased monotonically with insufflation pressure.

Table 1 shows the parameters estimated by curve fitting Eq. 1 for each animal. p_0_ ranged between 1.1 and 2.5 hPa. The IAV_max_ ranged between 2.79 and 4.61 L. The expansion rate ranged between 0.1 and 0.15 hPa^−1^. In all subjects, the RMSE for curve fitting this model was below 0.035 L. The C_ab,stat_ ranged between 0.0318 and 0.364 L/hPa. Figure 2b shows the calculated C_ab,stat_ for all individual subjects.

**Table 1:** Individual parameters and root mean squared errors obtained from the curve-fitted equation

Subject #	p_0_ (hPa)	IAV_max_ (L)	λ (hPa^−1^)	RMSE (L)
1	1.1	4.52	0.14	0.034
2	2.0	4.61	0.11	0.021
3	2.4	3.89	0.11	0.034
4	2.5	3.6	0.13	0.023
5	2.1	3.99	0.10	0.024
6	2.4	2.79	0.12	0.007
7	2.3	3.21	0.13	0.035
8	2.0	3.91	0.10	0.013
9	1.5	3.78	0.14	0.018
10	1.9	3.09	0.15	0.021
Median (i.q.r)	2.0 (1.9–2.4)	3.84 (3.21–3.99)	0.12 (0.11–0.14)	0.022 (0.018–0.034)

p_0_, baseline pressure; IAV_max_, maximum intra-abdominal CO_2_ volume; λ, pressure expansion rate; RMSE, root mean squared error

### Abdominal oscillatory impedance and dynamic compliance

**Fig. 3:**
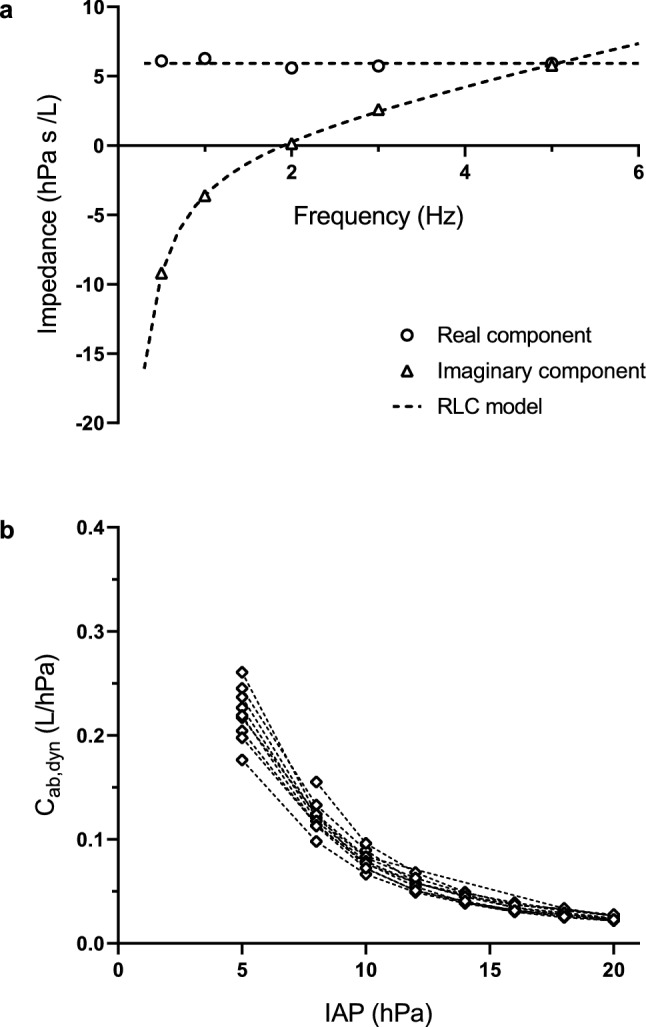
Example of impedance spectrum and estimated dynamic abdominal compliance. **a** Abdominal input impedance spectrum, resistance (○) and reactance (Δ) plotted vs frequency with the curve-fitted RLC-model (- -). **b** For each individual subject, the estimated dynamic abdominal compliance vs IAP (◊)

A total of 80 endoscopic oscillometry sequences were recorded. In one recording, mechanical ventilation interfered with the measurement. In three recordings the flow measurement was affected by occlusion of the insufflation tube. This resulted in a total of 76 analysed recordings. The magnitude squared coherence was above 96% for all impedance estimations. Figure 3a presents an example of the estimated Z_ab_ as well as the curve fit of the RLC-model.

Resistance ranged between 3.8 and 7.1 hPa s/L. Inductance ranged between 0.18 and 0.26 hPa s^2^/L. The C_ab,dyn_ ranged between 0.0216 and 0.261 L/hPa. In all subjects, C_ab,dyn_ decreased monotonically with IAP increments. The calculated RMSE was below 0.72 hPa s/L for all RLC-model fits. Figure 3b shows the C_ab,dyn_ curves for all individual subjects.

### Comparison between static and dynamic abdominal compliance

**Fig. 4:**
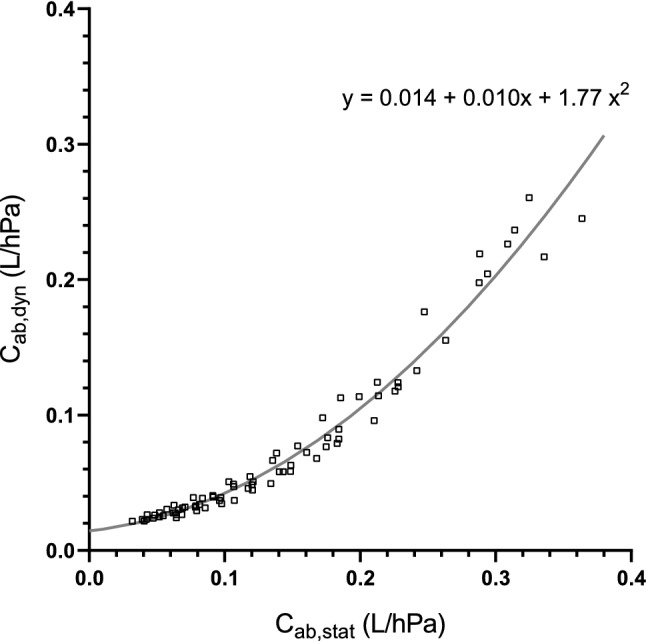
The correlation between dynamic and static compliance. Paired measurements of C_ab,stat_ and C_ab,dyn_ (□) and polynomial regression line (─), the adjusted R-squared was 97.1%

The relationship between static and dynamic abdominal compliance was analysed for a total of 76 pairs of measurements. Figure 4 reports the correlation plot and resulting polynomial regression line *y* = *0.014* + *0.010 x* + *1.77 x*^*2*^ for all measurement pairs. The calculated adjusted R-squared was 97.1%.

## Discussion

This study demonstrates that endoscopic oscillometry can be used to monitor changes in abdominal compliance during laparoscopy in a porcine model. A custom insufflation device was developed to determine C_ab_ from endoscopic oscillometry measurements obtained through the insufflation circuit. With the custom-built insufflation device, the chosen endoscopic oscillometry frequencies, peak-to-peak amplitude and signal processing methods, it was possible to obtain reliable estimations of C_ab_. This was validated by comparing endoscopic oscillometry to CT-based compliance measurements in a porcine laparoscopy model, showing a high degree of correlation.

The high adjusted R-squared (97.1%) proves that C_ab,dyn_ is a good predictor for monitoring changes in C_ab,stat_. It is important to realize that C_ab,dyn_ is a compound parameter that includes all tissues forming the boundaries of the abdominal cavity. Figure 4 shows that the relation between static and dynamic abdominal compliance is non-linear and C_ab,dyn_ underestimates C_ab,stat._ The non-linear relationship between the two measures for C_ab_ can be explained by the viscoelasticity of the tissues surrounding the abdominal cavity. In a viscoelastic model of the tissues, the measured compliance would depend on the rate of change in pressure. As a consequence, the determination of abdominal compliance differs between static and dynamic conditions. The high adjusted R-squared value indicates that the estimated coefficients can be used to compensate for this difference and allows conversion between C_ab,stat_ and C_ab,dyn_.

In this study, C_ab_ was investigated in a porcine model. The range of C_ab,stat_ that was found (0.03—0.37 L/hPa) is similar to C_ab_ found in human studies. Several studies in adult humans [11, 16, 27] have described the pressure–volume relationship of the abdominal cavity. McDougal et al. [16] (n = 41) and Abu-Rafea et al. [11] (n = 100) measured up to 40 hPa (30 mmHg) and found IAVs up to 9.8 L. In the summarized data presented in these two studies the C_ab_ ranges between an estimated 0.06 and 0.31 L/hPa. This is in line with a more recent study by Mulier et al. [27] (n = 100) in which C_ab_ ranged between an estimated 0.18—0.25 L/hPa. Only two studies, performed in a porcine model, showed values outside the C_ab_ range found in this study. The study by Vlot et al. [28] presented a lower range of 0.02—0.06 L/hPa, which can be explained by the low weight (± 6 kg) of the investigated porcine model. The study by McDougal et al. [16] reported a range of 0.02—0.12 L/hPa. Unfortunately, the weight of the porcine model used in their study was not reported and only calculated volumes were included.

To determine C_ab,stat_, our study utilized a non-linear model to describe the measured pressure–volume relationship. The low overall RMSE of less than 0.035 L/hPa in all subjects indicates that this model provides reliable estimations of C_ab_ in L/hPa over the entire pressure range of 5—20 hPa (3.75—15 mmHg). With a range exceeding 12 mmHg of insufflation pressure, this expands the concept of the linear model described by Mulier et al. [27]. The empirical equation allows non-linearity through the addition of expansion rate as a factor. The non-linear equation utilizes several mechanical parameters that allow comparison to previous literature. The mean value for p_0,_ 2 hPa, is close the value of 1.7 hPa observed by McDougal [16], but is lower than the value of 7.3 hPa observed by Mullier [27]. The range of estimated IAV_max_ values was large, especially when regarding the homogeneity of the porcine model. However, this range was similar to the range of measured IAVs at an IAP of 20 hPa (15 mmHg), suggesting accurate estimation. The expansion rate parameter was introduced in this study, hence it could not be compared to existing literature.

Endoscopic oscillometry can detect changes in C_ab_ during the creation of pneumoperitoneum, which can be used to individualize insufflation pressures. The custom-built insufflation device was developed to allow the application of the endoscopic oscillometry signal through the main lumen of a trocar. This measurement configuration requires no additional abdominal access or tubing. The high signal coherence indicated that the peak to peak amplitude (3 hPa) of the applied pressure oscillation was sufficient for an accurate estimation of Z_ab_, and resulted in barely discernible oscillations of the abdominal wall. We found that frequencies below 0.5 Hz are more sensitive to changes in abdominal mechanics due to CO_2_ insufflation, yet required a longer respiratory pause for measurements. Additionally, lower frequencies are more prone to trigger the mechanical ventilator to compensate for the applied pressure oscillations. At higher frequencies, endoscopic oscillometry is less sensitive to changes in C_ab_ because the inertial properties of the gas in the insufflation tube will affect Z_ab_ [29]. The results obtained in this porcine model for laparoscopy suggest that the range of frequencies used in this investigation (0.5 to 5 Hz) can be applied to measure similar compliance ranges in humans.

For comparison, both the CT and endoscopic oscillometry measurements were recorded during an end-expiratory pause at PEEP. Mechanical ventilation causes movement of the diaphragm, which is likely to change C_ab_. In addition, it is known that weight, body mass index (BMI), the use of NMB, age and history of pregnancy can affect tissue elastance and thereby C_ab_ [10–12]. In clinical application, these factors could add to the considerable natural variation in C_ab_ that was found in the homogeneous porcine model in this study. Therefore, the applicability of endoscopic oscillometry in clinical practice and the ability to monitor C_ab_ in patients of different sizes should be verified in a clinical trial. Such a clinical trial could provide more insight into the natural variation of C_ab_ between individuals and could be used for an improved mathematical RLC-model for the investigation and/or compensation of the interaction with mechanical ventilation.

Intraoperative and objective measurements of individual abdominal compliance could improve outcome of laparoscopy by allowing the surgeon to limit insufflation pressures and prevent excessive tissue stress due to overdistension of the abdominal cavity. Although useful for confirming the creation of a pneumoperitoneum, palpation of the abdomen is not suitable for determining individual abdominal compliance. Thus far, objective measures of C_ab_ have only been feasible in experimental settings. Although CT and MRI have been used to provide accurate and repeatable measurements in experimental research, their added value is negated by the drawbacks of these techniques. Interference with the procedure, the additional time added to the surgery and the presence of ionising radiation or strong magnetic fields prevent their routine use in clinical practice. Endoscopic oscillometry is a novel method that provides quantitative measurements of C_ab_ without interfering with the surgical workflow. This first evaluation of endoscopic oscillometry showed that it is feasible and accurate for monitoring changes in C_ab_ during laparoscopy.

Further development of endoscopic oscillometry should aim to provide surgeons with a tool for monitoring C_ab_ during the creation of pneumoperitoneum. The most simple implementation would entail a real-time plot of C_ab_ during stepwise insufflation, allowing the surgeon to visually detect the deflection point in the curve. Endoscopic oscillometry can be used to chart abdominal compliance during the stepwise creation of a pneumoperitoneum within the clinically acceptable timespan of 2 to 3 min. To avoid a high starting pressure, endoscopic oscillometry should be started at a pressure that does not provide sufficient surgical workspace. When incorporated into a surgical insufflator, real-time monitoring of the compliance curve can provide an easy to use and objective method for adhering to the surgical guidelines that advise to use the lowest possible IAP [2]. The effects of weight, BMI, age and history of pregnancy onto C_ab_ can be quantified using endoscopic oscillometry. Real-time monitoring of C_ab_ can be used to guide the optimization of surgical conditions using NMB and body positioning. These insights could allow personalization and improvement of treatment during minimal access surgery.

### Conclusions

Endoscopic oscillometry allows real-time monitoring of the changes in abdominal compliance during laparoscopic surgery in a porcine model. This is achieved by applying small oscillations in insufflation pressure and calculating abdominal compliance from the resulting pressure and flow in the insufflation circuit. Feasibility was demonstrated in a porcine model and validated against compliance derived from CT imaging. By monitoring abdominal compliance, the negative effects of CO_2_ insufflation can be reduced by preventing the application of excessive pressures. Clinical application of endoscopic oscillometry could provide surgeons with a practical tool for monitoring abdominal compliance.
